# The TEAM trial: Safety and efficacy of endovascular treatment of unruptured intracranial aneurysms in the prevention of aneurysmal hemorrhages: A randomized comparison with indefinite deferral of treatment in 2002 patients followed for 10 years

**DOI:** 10.1186/1745-6215-9-43

**Published:** 2008-07-16

**Authors:** Jean Raymond, Andrew J Molyneux, Allan J Fox, S Claiborne Johnston, Jean-Paul Collet, Isabelle Rouleau, the TEAM collaborative group

**Affiliations:** 1 TEAM coordinating centre, Interventional Neuroradiology Research Unit, Department of Radiology, CHUM Notre-Dame Hospital, 1560 Sherbrooke east, Pavilion Simard, room Z12909, Montreal, QC, H2L 4M1, Canada; 2 Oxford Neurovascular & Neuroradiology Research Unit, Level 6, West Wing, John Radcliffe Hospital, Headley Way, Oxford, 0X3 9DU, UK; 3 Department of Medical Imaging, Sunnybrook Health Sciences Centre, 2075 Bayview Avenue, room AG31b, Ontario, M4N 3M5, Canada; 4 UCSF Neurovascular Disease and Stroke Centre, University of California at San Francisco, 505 Parnassus avenue, San Francisco, CA, 94143-0114, USA; 5 Centre for Healthcare Innovation and Improvement, University of British Columbia, 4480 Oak Street, room E414A, Vancouver, BC, V6H 3V4, Canada; 6 Centre de Neurosciences de la Cognition, Département de Psychologie, UQAM, Box 8888, Succursale Centre-Ville, Montreal, QC, H3C 3P8, Canada

## Abstract

The management of patients with unruptured aneurysms remains controversial. Patients with unruptured aneurysms may suffer intracranial haemorrhage, but the incidence of this event is still debated; endovascular treatment may prevent rupture, but involves immediate risks. Hence, the balance of risks and benefits of endovascular treatment is uncertain. Here, we report the design of the TEAM trial, the first international, randomized, controlled trial comparing conservative management with endovascular treatment. Primary endpoint is mortality and morbidity (modified Rankin Score ≥ 3) from intracranial haemorrhage or treatment. Secondary endpoints include incidence of hemorrhagic events, morbidity related to endovascular coiling, morphological results, overall clinical outcome and quality of life. Statistical tests compare between probabilities at 5- and 10-years of 1/mortality from haemorrhage related to the lesion, excluding per-operative complications; 2/mortality from haemorrhage or from complications of treatment; 3/combined disease or treatment related mortality and morbidity in the absence of other causes of death or disability. The study will be conducted in 60 international centres and will enrol 2,002 patients equally divided between the two groups, a size sufficient to achieve 80% power at a 0.0167 significance to detect differences in 1) disease or treatment-related poor outcomes from 7–9% to 3–5%; 2) overall mortality from 16 to 11%. Duration of the study is 14 years, the first three years being for patient recruitment plus a minimum of 10 years of follow-up. The TEAM trial thus offers a means to reconcile the introduction of a new approach with the necessity to acknowledge uncertainties.

Current Controlled Trials ISRCTN62758344

## Background

The care of patients with unruptured aneurysms has been described as the most vexing scientific question confronting neurosurgeons, neurologists and interventional neuroradiologists [1,2]. The best management of patients with asymptomatic intracranial aneurysms is currently uncertain. The prevalence of intracranial aneurysms has been estimated at 1 to 2% of the adult population but with the increasing availability of non-invasive imaging of the brain in an aging population, unruptured aneurysms are increasingly being discovered [3-5].

Most aneurysms remain asymptomatic until the day they rupture, an event that occurs with an annual incidence of 8–10/100,000 in the overall population [3-5]. Subarachnoid haemorrhage (SAH) is associated with a high morbidity and mortality (45–75%) despite the advances of modern surgical and medical management [6,7]. Thus a preventive treatment strategy is appealing. The annual risk of bleeding from an unruptured aneurysm is debated, but most series and meta-analysis have reported a small annual risk, between 0.5–2% [4-7] with major morbidity or death affecting up to 60% of those patients with eventual ruptures [7]. Any preventive treatment should therefore be very safe. Endovascular treatment can prevent rupture but involves immediate risks to the patient [8-13]. Furthermore, successful treatment does not eliminate all risks [14,15].

The efficacy of endovascular treatment in the prevention of SAH remains unknown [16,17]. An international randomized trial has shown that endovascular treatment is safe and effective, and does improve the outcome of patients treated after subarachnoid haemorrhage as compared to surgical clipping, but this finding cannot be extrapolated to unruptured lesions [18].

Hence, the balance of the risks and benefits of coiling is uncertain. Nevertheless coiling of unruptured aneurysms is becoming the most frequent procedure performed in endovascular centres [19].

Prevention is justified when risks of treatment are low and when benefits have been supported by valid trials. Patients with unruptured aneurysms have never been the subjects of a randomized trial. While medicine only has an obligation of means, prevention has an obligation of results, because prevention only offers potential benefits and exposes healthy individuals to a certain risk [20]. The clinical dilemma: "Are patients with unruptured aneurysms best managed conservatively until definite indications arise or with endovascular treatment" can only find a resolution by resorting to the rigorous methodology of the randomized clinical trial [21-27].

We report the design of the TEAM trial (Trial on Endovascular Aneurysm Management), the first and currently only randomized trial on the management of unruptured aneurysms eligible to endovascular treatment.

## Overview of study design

TEAM is an international, randomized, multicenter, controlled trial comparing the combined mortality and morbidity (modified Rankin Scale (mRS) ≥ 3) from intracranial haemorrhage or treatment in patients with unruptured aneurysms treated by conservative management (or deferral of treatment for 10 years or until definite indications are thought to have arisen) as compared to endovascular coiling.

The study is designed as a pragmatic trial. All candidates for endovascular treatment of one or more unruptured intracranial aneurysms are potential participants. Unruptured aneurysms may be recently discovered or prevalent. If they accept, subjects will be randomized to one of the two arms of the trial: Conservative management or endovascular management.

Both groups will be advised to obtain medical treatment for hypertension if necessary and will receive counselling for behavioural risk factor modelling (smoking or excessive drinking) when indicated. A non-invasive (MRA or CTA) or catheter angiogram and a baseline CT-scan or MRI of the brain are required to enter the study. These studies should demonstrate the unequivocal presence of a saccular aneurysm ≥ 3 mm treatable by endovascular methods. A catheter angiogram is required if there is doubt. Imaging studies will be reviewed centrally. Both treatments will be standardized. Patients will be followed similarly for a minimum of 10 years.

The study will be conducted in 60 international centers. The entire study will enrol 2002 patients equally divided between the 2 groups, a size sufficient to achieve 80% power at a 0.0167 significance to detect differences in 1/disease or treatment-related poor outcomes from 7–9% to 3–5% as judged by an independent committee masked to treatment allocation; 2/overall mortality from 16% to 11%. The duration forecast for the study is 14 years, the first 3 years being for patient recruitment plus a minimum of 10 years of follow-up.

Secondary end points will include the incidence of hemorrhagic events in both groups, the morbidity related to endovascular coiling, morphological results as assessed by non-invasive imaging at 5 and 10 years, overall clinical outcome (morbidity and mortality) at 5 and 10 years, quality of life assessment (SF-36), and the level of distress caused by the knowledge of the hemorrhagic risk using the HADS questionnaire.

### Primary objective

The primary objective of the trial is to answer the question: Do patients with eligible unruptured aneurysms have a better clinical outcome at 10 years with endovascular or conservative management of their lesions (deferral of treatment until definite indications are thought to have arisen)?

Clinical outcome is defined as combined morbidity (modified Rankin scale 3 or more) and mortality from intracranial haemorrhage or from treatment (as judged by an independent committee unaware of treatment allocation).

### Secondary objectives

The secondary objectives include assessments of the 5 and 10-year risks of haemorrhage of patients of both groups, of the overall morbidity and mortality, of the safety and efficacy of endovascular treatment, of the morphological evolution of lesions at 5 and 10 years (stability of occlusion results of treatment and rate of growth of untreated lesions as judged centrally), a comparison between quality of life measures between the 2 groups, as well as an assessment of the psychological impact of harbouring treated or untreated unruptured aneurysms.

### Planned trial interventions

#### a. Conservative management

For patients randomized to the "conservative" arm, invasive treatment is deferred for at least 10 years or until definite indications are thought to have arisen (progressive symptoms from mass-effect, haemorrhage or symptoms/signs suggesting "impending" rupture such as IIIrd nerve palsy). Such events will be managed appropriately and reported as "adverse events" within 48 hrs to the appropriate committees. Certain centres may follow aneurysms by periodic imaging, and could act upon demonstration of "growth". The trial does not require such actions. These "growths", which we expect to be rare, will be reported. The final outcome, including treatment, considered within a "conservative strategy" will be included in the primary analysis.

#### b. Endovascular treatment

Endovascular treatment with coils will be performed within 1 month of randomization, according to current standards of practice, and under general anaesthesia, systemic anticoagulation and antiplatelet therapy. Lesions may recur at follow-up and physicians may elect to retreat early recurrences. Re-treatments will be registered, and recorded on Case Report Forms (CRFs) along with potential complications, but are considered intrinsic to "endovascular management", within the same entry. Many patients, including those with a family history, present multiple unruptured aneurysms (20–40%), sometimes associated with a previously treated, ruptured aneurysm (25%). It is common, and in fact an integral part of "endovascular management" to treat dominant lesions or those felt to be at higher risk and observe smaller ones or those felt to entail minor risks. We do not wish to exclude such patients since they are a most frequent indication for a preventive intervention. Rupture of an untreated lesion may be difficult to differentiate from bleeding of a treated one in the same patient, but this distinction is not pertinent since our main objective is to assess the benefits of endovascular management for the patient and not the efficacy of coiling of one aneurysm.

### Treatment allocation

Patients will be allocated to one of two groups: a) conservative management or deferral of treatment for 10 years or until indications have arisen or b) endovascular treatment, using a centralized, minimisation procedure taking into account patient and aneurysm characteristics to ensure a balanced distribution between groups; minimisation criteria have been set to the following (decreasing hierarchical order): 1/type of presentation at diagnosis: patients with a previously treated ruptured aneurysm or not ; 2/aneurysm location: anterior versus posterior circulation; 3/aneurysm dimensions: aneurysm greater than or equal to 7 mm or not.

After on-line verification of the inclusion criteria the treatment will be allocated to the patient; randomization is through a web-based electronic Data Management system that is available 24 hours a day, seven days a week.

### Selection of subjects

The goal of the trial is to include any patient with unruptured aneurysms considered a candidate for endovascular management. To take into account ease of recruitment, feasibility, generalizability, and ethical considerations, we have kept entry criteria to a minimum (see Table 1). Scientific and ethical considerations forbid a rigid a priori set of criteria for equipoise. To enrol a patient in such a trial necessitates the acknowledgment that we do not know for certain what is the best management of his or her condition. In patients in whom risks of treatment may appear reasonable, the risks of future haemorrhage may also be modest. Another lesion presenting with characteristics raising concerns in relation to haemorrhage may also carry increased immediate procedural risks. Although we favour a team approach regarding therapeutic decisions, the final judgment on equipoise will rest upon the TEAM-physician consulted by the patient. Reasons for not using ISUIA subgroups results for selection criteria are explained in [26]. Because some limits were thought to be indicated, very small (<3 mm) and giant aneurysms (> 25 mm) were excluded because of increased risks of perforation and poor efficacy of coiling respectively. Patients with multiple aneurysms, including those that were successfully treated after a rupture, perhaps associated with an increased incidence of ruptures of associated unruptured lesions, are patients for whom the research question is crucial. Thus they can be recruited provided the ruptured aneurysm is fully excluded from the circulation, to prevent confounding the management of ruptured and unruptured aneurysms. Other exclusion criteria concern the need to exclude other causes of haemorrhage, patients that cannot be assessed for the primary endpoint at the end of the observation period, and patients that cannot be managed endovascularly.

**Table 1:** Selection criteria

**Inclusion criteria**	
a.	At least one documented subarachnoid aneurysm, never ruptured
b.	Patient aged 18 or older
c.	Life expectancy more than 10 years

**Exclusion criteria**	

a.	Patients with recent (less than 3 months) intracranial haemorrhage
b.	Lesion characteristics unsuitable for endovascular treatment
c.	Patients with a single extradural aneurysm
d.	Aneurysms <3 mm or giant aneurysms (≥25 mm)
e.	Patients with a poor outcome (Rankin scale ≥3) after the rupture, surgical or endovascular treatment of another aneurysm
f.	Patients with incompletely treated aneurysms that have previously ruptured
g.	Patients with associated arteriovenous malformations
h.	Patients with new severe progressive symptoms in relationship with the aneurysm (sudden onset, severe persisting headaches suggestive of impending rupture, third-nerve palsy, mass-effect)
i.	Patients with previous intracranial haemorrhage from unknown aetiology
j.	Patients with multiple unruptured aneurysms in whom surgical clipping of one or many aneurysms is planned in addition to endovascular management
k.	Patients with absolute contraindications to anaesthesia, endovascular treatment, or administration of contrast material, including low-osmolarity agents or gadolinium
l.	Pregnant patients
m.	Patients unable to give informed consent

### Frequency and duration of follow-up

Endpoint events are expected to occur with an incidence of 0.5–2% per year in the "control group", with a morbidity/mortality of 60% when they occur. Treatment aims at long-term protection. This requires a relatively long follow-up, proposed as a minimum observation period of 10 years. However, recruitment needs to be done within 3 years for this trial to be completed within a reasonable time frame. Subjects from both trial arms will be followed at one, six, twelve months, and yearly for 10 years. Telephone contact will be performed at 6 month intervals to minimize losses to follow-up. Clinical assessments will comprise: 1) examination by an independent physician at discharge (including an mRS), and by the treating physician at 1 and 6 months and yearly thereafter for 10 years; 2) evaluation by an independent and blinded interviewer according to the mRS, the MoCA, SF-36, EQ-5D (Euroqol) and HADS questionnaires at the time of recruitment, and at one, 5 and 10 years.

### Primary endpoint

The primary outcome, disease or treatment-related morbidity or mortality, will be defined as the number of patients with a mRS ≥3, from hemorrhagic events, ischemic stroke from SAH or treatment, or operative complications, for intent-to-treat populations at 1, 5 and 10 years.

The specific hypothesis is: The 10-year combined mortality and morbidity (M/M) related to unruptured aneurysms observed in the conservative group will be decreased from 8% to 4% (M/M of treatment and hemorrhagic events despite treatment as expressed by a mRS ≥ 3) with endovascular treatment. Like any trial involving a long follow-up period there will be a number of unrelated events leading to death or morbidity; to keep sample size reasonable, endpoints have to be adjudicated as disease or treatment-related, or disease or treatment-unrelated [28].

### Outcome measurements

#### a. Modus operandi

At the onset of the trial, a Clinical Events Committee (CEC) has defined the types of events to be adjudicated as disease or treatment-related, established criteria for those events and set the algorithm to be followed in order to classify a clinical event. The CEC proposal will be vetted by the Data Safety and Monitoring Committee (DSMC). The Endpoint Review Committee (ERC), following the algorithm set by the CEC and approved by the DSMC and operating blindly with respect to treatment allocation, will adjudicate events based on censored clinical data documenting the event. Censoring will include digital manipulation of radiology imaging data in order to prevent adjudicators from being able to guess group allocation; it will be performed by a dedicated Image Masking Center, in Edinburgh.

#### b. Definitions

Criteria for the following events were set : intracranial haemorrhage, aneurysm-related stroke, complications of adjunct treatment to endovascular procedure and complications from mass effect due to the aneurysm. Other types of events will be considered unrelated to the aneurysmal disease or its treatment. Below, some categories are more explicitly defined.

Treatment-related mortality or morbidity is defined as any event leading to mortality or disability (mRS ≥3) occurring within 2 months of randomisation or within 1 month of the endovascular procedure (delayed mortality from operative morbidity is considered "operative" even beyond 1 month) or due to complications of adjunct treatment to endovascular procedure.

Disease-related morbidity or mortality is defined as any event leading to mortality or disability (mRS ≥3) occurring within 2 months of randomisation, or secondary to hemorrhagic events, ischemic strokes related to the aneurysm, or mass effect, for the remainder of the follow-up period.

Hemorrhagic event is defined as 1) cross-sectional imaging (CT or MRI) evidence of intracranial bleeding or 2) acute headache and a lumbar puncture positive for haemorrhage or 3) acute third-nerve palsy if the aneurysm is in the vicinity or 4) sudden death preceded by severe headaches or 5) intracranial bleeding proven by post-mortem CT-scanning or autopsy or 6) unexplained sudden death.

Lethal hemorrhagic event is any mortality subsequent to intracranial haemorrhage or its treatment.

"Impending rupture" in the conservative management group. Patients with symptoms of impending rupture during follow-up (see above in exclusion criteria), a relatively rare event that could justify urgent endovascular or surgical treatment will be considered "failures" of conservative management; "impending rupture" will thus be grouped with "hemorrhagic events" in Kaplan-Meier analyses of "survival without haemorrhagic event". However, such cases will not be included in the primary outcome of the conservative management group unless they exhibit mortality or morbidity (mRS ≥ 3) from justified treatment. This is warranted in a trial comparing elective invasive treatment with "conservative management", since performing interventions for clinical indications as they occur is considered an integral part of "conservative management".

#### c. Primary outcome

The primary outcome, disease or treatment-related morbidity or mortality, will be defined as the number of patients with an mRS ≥3 from events classified as related to the aneurysm or its treatment, for intent-to-treat populations at 1, 5 and 10 years. To minimize bias the interviewers administrating the mRS and the committee adjudicating the relation to the disease or treatment (the ERC) will not be aware of group assignation.

#### d. Secondary outcomes

We will compare Kaplan-Meier (KM) curves of "survival without hemorrhagic event" and "survival without hemorrhagic death" between the two groups for intent-to-treat populations. In addition, we will repeat the same analyses after removing cases of "impending rupture" from hemorrhagic events and deaths.

To measure morbidity/mortality of endovascular treatment, all treated patients will be assessed by an independent neurologist before discharge and all severe adverse events (SAE) will be reviewed by the Adverse Event Committee (AEC) to assure quality control on a case-by-case basis. SAE, those that are life threatening or leading to prolonged hospitalizations, will be reported within 48 hours to the data coordination centre that will transmit the information to the DSMC. Severe procedure-related complications will be reviewed in detail on a case by case basis to assess if they were unavoidable, caused by a technical error, or a breech in standard care. The DSMC will then recommend an on-site visit or suspend recruitment in individual centres if appropriate.

The death rate will be recorded for the intent-to-treat analyses. It will be obtained by dividing the number of deaths by the number of patients in each group. The number and the severity of morbid events will be recorded for each patient. The mRS will be assessed blindly at 1, 5 and 10 years.

Morphological results after treatment, at 6 months, and at 5 and 10 years will be categorized using a validated classification into 3 classes [14]. The number of patients showing a recurrence, defined as aneurysmal growth or recanalization, at follow-up imaging for all patients followed at 5 and 10 years will determine the recurrence rate. Interim angiographic results (invasive or non-invasive) at 6 months and 5 years, if there is no re-treatment, will help determine a relationship between recurrences and hemorrhagic episodes in the treated group, if there is any.

The incidence of aneurysmal "growth" as documented by non-invasive imaging in the group "treated conservatively" may provide additional data, but is of unknown significance. Images from all patients, at baseline, at the end of treatment (for endovascular group), at 6 months, and at 5 and 10 years will be analyzed centrally. An Imaging Committee will determine aneurysm size for all patients and criteria for "recurrences" and "aneurysmal growth" on follow-up imaging studies. Aneurysms will be compared over time to see if growth can be observed which will be referred to as aneurysm "instability", or remain the same and considered "stability". Aneurysm growth in the control group and recurrences in the treated group will be a surrogate endpoint that could predict greater future risk of rupture.

A set of quality of life and anxiety/depression measures will be collected with the 36-Item Short-Form Health Survey [29-33], the EQ-5D (Euroqol) and the HADS questionnaires at baseline, and at 1, 5 and 10 years. The SF-36 and EQ-5D is normally completed by the patients but the present study will ensure the presence of interviewers to ensure adequate completion and comprehension of the questions; interviewer will ignore patient group assignment. We will compare the absolute scores of both group, the number of poor results (cut-off points of 7 for HADS and 40 for SF-36) as well as the number of patients with worsened scores with time between groups.

All patients will be assessed with the MoCA [34-37] at baseline, and at 1, 5 and 10 years. This instrument was preferred to the more widely used MMSE because it includes some measures of executive functions (verbal fluency and shortened version of Trail Making Test, part B) as well as measures of naming abilities (animal naming) which are lacking in the MMSE. In addition, for the assessment of visuospatial abilities, the copy of the two embedded geometric figures (from MMSE) was replaced by cube copying and by clock drawing under command (with time set at 10 after 11), which has been shown to be highly sensitive to brain dysfunction [38]. Because cognitive functions could be altered with treatment, but abnormalities may not be discovered by standard examinations and using trial tests, we will also perform detailed neuropsychological assessments in 100 consecutive patients of both groups at baseline and 6 months after randomization in dedicated centres. Because of their high sensitivity, tests of executive functions, attention and episodic memory will be administered. In agreement with the NINDS recommendations, the following tests will be administered in addition to the MoCA: the HVLT (12 words from 3 categories learned over three trials with delayed recall and recognition), copy and delayed recall of the Rey complex figure, BNT (confrontation naming of 60 pictures), Digit Symbol subtest of the WAIS-III, COWAT (a letter fluency test), Trail Making Test A and B. These measures are similar to the ones examined following surgical clipping [39].

### Sample size calculation, planned analyses and control of error

As in any trials of hazardous surgery, early risks may be followed by later benefit. Therefore the hazard ratio will be unfavourable during the recruitment years while interventions are being performed [28,40]. Although more sensitive tests will be performed to assess some hypotheses (Kaplan-Meier methods for hemorrhagic events and mortality), we used two-sided log rank tests to estimate sample size. We took into account patients lost for analyses, and adjusted the alpha for interim analyses. The total size of the population would be approximately 2002 (taking into account 12% (or 240) patients being lost for analysis (8–10% of unrelated deaths [7] and 1–2% lost to follow-up [28]), based on the following hypotheses: The overall benefit of endovascular management could be demonstrated with a total of 1688 patients, which is the number to achieve 80% power at a 0.0167 significance level (to account for 1–5-year interim analyses) to detect a difference of 0.04 (hazard ratio: 0.4896) between the null hypothesis that both proportions are 0.08 (the disease/treatment-related morbidity/mortality reaches 8% at 10 years) and the alternate hypothesis that the proportion of the endovascular group is 0.04. These estimates are compatible with 1) the recent metanalysis by Wermer [4] for the risks of observation, with a 60% morbidity/mortality associated with a 1.2% yearly hemorrhagic rate and 2) with the Atena registry on the morbidity and mortality of endovascular treatment of unruptured aneurysms in French centres [13]. Increasing the size of the population to 2002 was elected to allow 1) to verify a significant difference under the same conditions between 7–9% in the conservative group to 3–5% in the endovascular group (Table 2) and 2) to achieve 80% power at a 0.0167 significance level to detect a difference of 0.05 (between 0.84 and 0.89) in the proportions surviving in groups 1 and 2 (difference in overall mortality; hazard ratio: 0.6684; proportion lost to follow-up 0.02; Table 3). A significant difference in the overall mortality, an outcome resistant to bias, could offer another convincing evidence for a benefit.

**Table 2:** Sample sizes to detect a significant difference in disease or treatment-related combined morbidity/mortality. Numeric Results with Proportion Lost to Follow Up = 0.1200

						**Hazard Two-sided**		
**Power**	**N**	**N1**	**N2**	**S1**	**S2**	**Ratio**	**Alpha**	**Beta**

0.8007	1332	666	666	0.9000	0.9500	0.4868	0.0167	0.1993
0.8006	872	436	436	0.9000	0.9600	0.3875	0.0167	0.1994
0.8006	602	301	301	0.9000	0.9700	0.2891	0.0167	0.1994
**0.8004**	**1947**	**974**	**973**	**0.9100**	**0.9500**	**0.5439**	**0.0167**	**0.1996**
0.8002	1168	584	584	0.9100	0.9600	0.4328	0.0167	0.1998
0.8007	757	379	378	0.9100	0.9700	0.3230	0.0167	0.1993
0.8002	3223	1612	1611	0.9200	0.9500	0.6152	0.0167	0.1998
0.8002	1688	844	844	0.9200	0.9600	0.4896	0.0167	0.1998
0.8003	1001	501	500	0.9200	0.9700	0.3653	0.0167	0.1997
0.8000	6717	3359	3358	0.9300	0.9500	0.7068	0.0167	0.2000
0.8002	2758	1379	1379	0.9300	0.9600	0.5625	0.0167	0.1998
**0.8004**	**1424**	**712**	**712**	**0.9300**	**0.9700**	**0.4197**	**0.0167**	**0.1996**

**Summary Statements: **A two-sided log rank test with an overall sample size of 1688 subjects (of which 844 are in group 1 and 844 are in group 2) achieves 80% power at a 0.0167 significance level to detect a difference of 0.0400 between 0.9200 and 0.9600 – the proportions surviving in groups 1 and 2, respectively. This corresponds to a hazard ratio of 0.4896. The proportion of patients lost during follow up was 0.1200.

**Table 3:** Sample sizes to detect a significant difference in overall mortality. Numeric Results with Proportion Lost to Follow Up = 0.0200

						**Hazard Two-sided**		
**Power**	**N**	**N1**	**N2**	**S1**	**S2**	**Ratio**	**Alpha**	**Beta**

0.8002	1520	760	760	0.8200	0.8800	0.6442	0.0167	0.1998
0.8004	1089	545	544	0.8200	0.8900	0.5872	0.0167	0.1996
0.8003	2125	1063	1062	0.8300	0.8800	0.6861	0.0167	0.1997
0.8005	1437	719	718	0.8300	0.8900	0.6254	0.0167	0.1995
0.8001	3219	1610	1609	0.8400	0.8800	0.7332	0.0167	0.1999
0.8000	2002	1001	1001	0.8400	0.8900	0.6684	0.0167	0.2000
0.8001	5543	2772	2771	0.8500	0.8800	0.7866	0.0167	0.1999
0.8001	3025	1513	1512	0.8500	0.8900	0.7170	0.0167	0.1999
0.8000	12068	6034	6034	0.8600	0.8800	0.8476	0.0167	0.2000
0.8001	5194	2597	2597	0.8600	0.8900	0.7727	0.0167	0.1999
0.8000	46657	23329	23328	0.8700	0.8800	0.9179	0.0167	0.2000
0.8000	11271	5636	5635	0.8700	0.8900	0.8368	0.0167	0.2000

**Summary Statements: **A two-sided log rank test with an overall sample size of 2002 subjects (of which 1001 are in group 1 and 1001 are in group 2) achieves 80% power at a 0.0167 significance level to detect a difference of 0.0500 between 0.8400 and 0.8900 – the proportions surviving in groups 1 and 2, respectively. This corresponds to a hazard ratio of 0.6684. The proportion of patients lost during follow up was 0.0200.

**Software: **PASS 2000, Power Analysis and Sample Size for Windows; NCSS, Kaysville, Utah

Thus the control of alpha error will be implemented by limiting interim analyses to two analyses once all patients have completed one and 5 years of follow-up. The final analysis will take into account multiplicity of testing for the primary endpoint as well as for overall mortality. All other secondary analyses will use a nominal P = 0.05. In order to describe how and when hemorrhagic events occur, analyses will include Kaplan-Meyer life-table methods to assess the 5- and 10-year survival, and survival without haemorrhage, among all those allocated immediate coiling (including the few who did not undergo it) and all those allocated deferral of any intervention (including the few who will eventually be operated on). The "survival" functions will be compared graphically and using a log-rank statistic. The main statistical tests will involve comparisons between the probabilities of mortality 1/from haemorrhage, excluding per-operative complications, 2/from haemorrhage or from complications of treatment, or 3/comparisons of the 5-year probabilities of combined disease/treatment-related mortality/morbidity, in the absence of other causes of death or disability. Descriptive statistics will be done on demographic variables and potential risk factors to compare the two groups at baseline. Means, standard deviations and range will be presented for quantitative variables such as size of aneurysms and frequency tables for categorical variables (such as number of patients with a previous history of SAH, or multiple aneurysms). Those statistics will be broken down by centre and by treatment arm. Comparability of the groups will be assessed through independent ANOVAs (quantitative data) or Mantel-Haentzel and χ^2 ^tests (categorical data). Assuming comparability of groups across centres, the primary outcome, disease or treatment-related combined morbidity/mortality for intent-to-treat populations will be compared between groups through a z-test for independent proportions at 1, 5 and 10 years. Similar analyses will be done for disease or treatment-related mortality. Secondary outcomes and overall morbidity will be compared between groups through independent t-tests (quantitative variables) or χ^2 ^statistics (categorical data). The analyses of neurological data at follow-up will control for baseline data using logistic regression, ANCOVA or Cox regression multivariate models. Finally, a logistic regression will be used to find variables capable of predicting haemorrhages or complications in both groups. The method planned is a stepwise forward with alpha <0.05 to enter a predictor. Possible predictors include the status of the aneurysm (previous history of SAH vs. unruptured only), size of the aneurysm (≥ 7 mm vs < 7 mm), location (posterior vs. anterior circulation aneurysms), angiographic results of endovascular treatment, as well as other baseline characteristics.

### Equipoise, randomization and ethical considerations

We cannot reliably compare the outcome following two treatment options without resorting to randomization. Genuine respect for subjects and human dignity requires that the research meet scientific norms of excellence (here randomization). The use of subjects can only be justified when the objective is to provide unbiased results and thus forward the knowledge regarding the disease or its treatment. Thus we want to reach generalizable knowledge but the physician's primary duty is to care for the individual patient. And this is where the difficulty is, an apparent opposition between generalizable knowledge and the care of the particular patient. This does not mean that we are bending the therapeutic obligation to current individuals to meet the scientific requirements that will provide knowledge to guide the treatment of future individuals. The research question concerns first and foremost our current patients, for whom no action has yet been proved beneficial. Thus randomization is not only a scientific solution to the problem of bias; it is also a practical way of assuring the best possible outcome for each individual patient. In the absence of evidence, by treating patients to prevent ruptures we could cause more harm than by not treating them. The best option for each individual is currently unknown. Until it can be asserted with confidence that patients need to be treated, because they do better with treatment than without, the "best" we can offer to our patient is a chance to be treated and thus to be protected from rupture of the aneurysm, and an equal chance not to be treated, and hence to be exempted from immediate treatment complications. Hence, when the uncertainty dominates, we offer randomization until the uncertainty can be replaced by reliable evidence. Now, how and when the reliability of evidence will be determined is a judgement to be delegated to an impartial and independent group of persons, the DSMC [25,26].

We have the moral duty to determine how appropriate our actions may be, actions that have so far only been guided by fear of the disease and faith in our technologies. We also have the duty to help the healthy individuals that we have put into difficult decisional contexts by our own technological progress, to understand that the uncertainty cannot be simply resolved. We have the professional responsibility not to act like if we knew.

Randomization is not giving up the decision to chance. It is to opt for a rational, responsible choice, to suspend judgment until there is evidence, to maximize chances of a benefit for each individual patient while we remain uncertain, and to act in a context that will promote knowledge and progress, in the respect of patient autonomy.

## Conclusion

Endovascular treatment is nowadays a standardized procedure that has routine applications in specialized centres. An objective assessment of its value in unruptured aneurysms is now necessary. A randomized trial can reconcile the introduction of a new approach with the necessity to acknowledge uncertainties, to scientifically assess potential benefits, and to assist healthy individuals alerted by the discovery of an ominous condition, in a controlled environment that respects and promotes their autonomy.

## Availability and requirements

The study website: 

## Abbreviations

BNT: Boston Naming test; CEC: Clinical Event Committee; COWAT: Controlled Oral Word Association Test; CRF: Case Report Form; CTA: Computed Tomography Angiography; DSMC: Data Safety and Monitoring Committee; EQ-5D: Euroquol (European quality of life); ERC: Endpoint Review Committee; HADS: Hospital Anxiety and Depression Scale; HVLT: Hopkins Verbal Learning Test; ISUIA: International Study of Unruptured Intracranial Aneurysms; KM: Kaplan Meier; MMSE: Mini Mental State Examination; MoCA: Montreal Cognitive Assessment; MRA: Magnetic Resonance Angiography; MRI: Magnetic Esonance Imaging; mRS: Modified Rankin Scale; SAH: Sub-Arachnoid Hemorrhage; SF-36: Short Form 36 (36 item questionnaire on Quality of Life); TEAM: Trial on Endovascular Aneurysm Management; WAIS: Wechsler Adult Intelligence Scale.

## Competing interests

The authors declare that they have no competing interests.

## Authors' contributions

JR, AJM, AJF, JSC, JPC, IR participated in the conception and design of the trial, in obtaining Canadian Institutes of Health Research (CIHR) funding and helped with drafting of the protocol.

## TEAM Collaborative group

### Steering committee

Pr Jacques Moret, Paris ; Dr Alejandro Berenstein, New York; Dr Herman Zeumer, Hamburg; Dr In Sup Choi, Boston; Dr Cameron McDougall, Phoenix; Dr Gabriel J. E. Rinkel, Utrecht; Pr Ling Feng, Beijing; Dr Julian Spears, Toronto; Dr Jean Raymond, Montreal; Dr Andrew Molyneux, Oxford; Dr S. Claiborne Johnston, San Francisco; Dr Isabelle Rouleau, Montreal; Dr Allan J. Fox, Toronto; Dr Jean-Paul Collet, Vancouver; Dr Yves Lepage, Montreal ; Antonieta Gasparini (CIHR, Ottawa); Guylaine Gevry, Ruby Klink and Marcia Loor, Montreal.

### Data Safety and Monitoring Committee

Pr Luc Picard, Nancy (Chair); Dr Michael Eliasziw, Calgary (clinical statistician); Dr Louise-Hélène Lebrun, Montreal (neurologist) ; Dr Gerald R. Winslow, Loma Linda (ethician); M. James Hosinec, Montreal (patient representative).

### Clinical Events Committee

Dr Charles Strother, Madison (Chair); Dr Karl-Fredrik Lindegaard, Oslo (neurosurgeon); Dr Daniel Roy, Montreal (neuroradiologist); Dr Sylvain Lanthier, Montreal (neurologist).

### EndPoint Review Committee

Dr Robert Coté, Montreal (neurologist); Dr Jeffrey Minuk, Montreal (neurologist); Dr Ariane Mackey, Quebec (neuroradiologist).

### Expert Committees

**Imaging Center: **Dr Allan J. Fox, Toronto; Dr Alain Weill, Montreal

**Data Preparation and Masking Center: **Dr Philip White, Edimburg

**Neuropsychology: **Dr Isabelle Rouleau, Montreal

**Patient Support Group: **Dr Maria Angeles de Miquel, Barcelona

### Participating Centres

See Table 4.

**Table 4:** Participating Centres.

**FRANCE**		
Angers	Hôpital Larrey	Pasco-Papon A.
Besançon	CHU Jean Minjoz	Bonneville JF.
Caen	CHU Côte-de-Nacre	Courtheoux P.
Clermont-Ferrand	Hôpital Gabriel Montpied	Chabert E.
Colmar	Hôpital Pasteur	Tournade A.
Créteil	Hôpital Henri Mondor	Gaston A; Blanc R.
Grenoble	Hôpital Albert Michalon	Le Bas JF.
Lille	Hôpital Salengro	Pruvo JP; Leclerc X.
Limoges	Hôpital Dupuytren	Chapot R.
Lyon	Hôpital Pierre Wertheimer	Turjman F; Lamy B; Tahon F.
Nancy	Hôpital Central	Bracard S; Anxionnat R.
Nantes	Hôpital Laennec	De Kersaint Gilly A; Desal H.
Paris	CH Sainte-Anne	Meder JF; Trystram D; Godon-Hardy S.
	Fondation Rothschild	Moret J; Piotin M; Spelle L; Mounayer C.
	Hôpital Saint-Joseph	Zuber M.
	Lariboisière	Houdart E.
	Pitié-Salpêtrière	Biondi A; Bonneville F; Jean B; Sourour N; Chiras J.
Reims	Hôpital Maison Blanche	Pierot L; Gallas S.
Saint-Etienne	Hôpital Bellevue	Manera L.
Suresnes	Hôpital Foch	Rodesch G.
Toulouse	Hôpital Purpan	Cognard C; Januel AC; Tall P.
Tours	Hôpital Bretonneau	Herbreteau D.
**UNITED KINGDOM**		
Bristol	Frenchway Hospital	Molyneux AJ.
Oxford	John Radcliffe Hospital	Byrne J; Kerr R.
Plymouth	Derriford Hospital	Adams W.
Birmingham	University Hospital	Lamin S.
Cardiff	University Hospital of Whales	Halpin S.
Edinburgh	Royal Infirmary Western General Hospital	White P; Sellar R.
Essex	Centre for Neurological Sciences	Chawda S.
Liverpool	The Walton Centre	Nahser H; Shaw D.
London	Kings College Hospital	Jeffree M.
	University College Hospital	Grieve J; Kitchen N.
Newcastle	General Hospital	Gholkar A.
Nottingham	Queens Medical Centre	Lenthall R.
Preston	Royal Preston Hospital	Patankar Tufail F.
Salford	Hope Hospital and Manchester Royal Infirmary	Hughes D; Laitt R; Herwadkar A.
Southampton	Wessex Neurological Centre	Millar J.
West Sussex	Brighton and Sussex University Hospital	Olney J.
**CANADA**		
Montréal	CHUM Hôpital Notre-Dame	Raymond J; Roy D; Guilbert F; Weill A.
	Montreal Neurological Institute	Tampieri D; Mohr G.
Québec	Hôpital Enfant-Jésus	Milot G; Gariépy JL.
Vancouver	Vancouver General Hospital	Redekop G.
Ottawa	Ottawa Hospital	Lum C.
Winnipeg	Health Sciences Center	Silvaggio J, Iancu D
Toronto	St Michael's Hospital	Marotta T.
**UNITED STATES**		
Chicago	Rush University Medical Center	Chen M., Lee V., Temes R.
Iowa	University of Iowa Hospitals and Clinic	Chaloupka J., Hayakawa M..
Houston	The Methodist Hospital	Klucznik RP.
Boston	Boston Medical Center – Boston University School of Medicine	Kase C., Lau H.,
New York	INN Beth Israel	Berenstein A; Niimi Y.
	Cornell Medical Centre	Gobin P.
	SUNY Downstate Medical Center	Mangla S.
Phoenix	Barrow Neurological Institute	McDougall C.
Charleston	Medical University of South Carolina	Turk A., Parker A.
Minneapolis	University of Minnesota Medical Center	Tummala R., Qureshi A.
**GERMANY**		
Dresden	Universitatsklinikum Carl Gustav Carus	Von Kummer R.
Hamburg	Universitatsklinikum Hamburg-Eppendorf	Zeumer. J; Fiehler H.
**ITALY**		
Milano	Ospedale Niguarda	Valvassori L., Boccardi E., Quillici L.
**NORWAY**		
Oslo	Rikshopitalet University Hospital	Bakke S.J; Kindergaard KF.
**POLAND**		
Warsaw	Instytute of Psychiatry and Neurology I Klinika Neurologiczna	Kobayashi A.
**SPAIN**		
Barcelone	Hospital Bellvitge	de Miquel M.A.
**BELGIUM**		
Gent	University of Gent Hospital	Defreyne L.
**BRAZIL**		
Rio Grande do Sul	Hospital de Clinicas de Porto Alegre	Stefani M.
**HUNGARY**		
Budapest	National Institute of Neurosurgery	Szikora I.; Kulcsar Z.
Miskolc	Borsod County Teaching Hospital	Lazar I.
